# Identifying Immune-Specific Subtypes of Adrenocortical Carcinoma Based on Immunogenomic Profiling

**DOI:** 10.3390/biom13010104

**Published:** 2023-01-04

**Authors:** Qiqi Lu, Rongfang Nie, Jiangti Luo, Xiaosheng Wang, Linjun You

**Affiliations:** 1 Biomedical Informatics Research Lab, School of Basic Medicine and Clinical Pharmacy, China Pharmaceutical University, Nanjing 211198, China; 2 Cancer Genomics Research Center, School of Basic Medicine and Clinical Pharmacy, China Pharmaceutical University, Nanjing 211198, China; 3 Big Data Research Institute, China Pharmaceutical University, Nanjing 211198, China; 4 Center for New Drug Safety Evaluation and Research, China Pharmaceutical University, Nanjing 211198, China

**Keywords:** adrenocortical carcinoma, immunological classification, clustering analysis, steroid hormones, tumor immune microenvironment

## Abstract

Background: The tumor immune microenvironment (TIME) of adrenocortical carcinoma (ACC) is heterogeneous. However, a classification of ACC based on the TIME remains unexplored. Methods: We hierarchically clustered ACC based on the enrichment levels of twenty-three immune signatures to identify its immune-specific subtypes. Furthermore, we comprehensively compared the clinical and molecular profiles between the subtypes. Results: We identified two immune-specific subtypes of ACC: Immunity-H and Immunity-L, which had high and low immune signature scores, respectively. We demonstrated that this subtyping method was stable and reproducible by analyzing five different ACC cohorts. Compared with Immunity-H, Immunity-L had lower levels of immune cell infiltration, worse overall and disease-free survival prognosis, and higher tumor stemness, genomic instability, proliferation potential, and intratumor heterogeneity. Furthermore, the ACC driver gene *CTNNB1* was more frequently mutated in Immunity-L than in Immunity-H. Several proteins, such as mTOR, ERCC1, Akt, ACC1, Cyclin_E1, β-catenin, FASN, and GAPDH, were more highly expressed in Immunity-L than in Immunity-H. In contrast, p53, Syk, Lck, PREX1, and MAPK were more highly expressed in Immunity-H. Pathway and gene ontology analysis showed that the immune, stromal, and apoptosis pathways were highly enriched in Immunity-H, while the cell cycle, steroid biosynthesis, and DNA damage repair pathways were highly enriched in Immunity-L. Conclusions: ACC can be classified into two stable immune-related subtypes, which have significantly different antitumor responses, molecular characteristics, and clinical outcomes. This subtyping may provide clinical implications for prognostic and immunotherapeutic stratification of ACC.

## 1. Introduction

Adrenal cortical carcinoma (ACC) is a rare but aggressive endocrine malignancy derived from the adrenal cortex. It has an annual incidence of 0.7–2.0 per million [1] and presents in most cases with signs and symptoms of adrenal steroid excess [2]. Currently, prognostic indicators for patients with ACC include tumor stage and tumor proliferation index, which are regarded as the strongest and most consistent prognostic markers [3]. To date, the widely used classification of ACC is based on the revised TNM classification by the European Adrenal Tumor Research Network (ENSAT) [4]. The current standard of care for unresectable or metastatic ACC is chemotherapy, radiation, and the adrenolytic drug mitotane, all of which are palliative [1]. Although unresectable or metastatic ACCs have an overall unfavorable prognosis, their outcomes are heterogeneous [5]. For example, based on four platforms’ datasets (DNA copy number, mRNA expression, DNA methylation, miRNA expression), The Cancer Genome Atlas (TCGA) network defined three molecular subtypes of ACC through Cluster of Cluster (CoC) analysis, namely CoC I, CoC II, and CoC III [6]. The subtype CoC I had the lowest rate of disease progression (7.0%), while another subtype, CoC III, had the highest rate of disease progression (96%).

Currently, immunotherapy, such as immune checkpoint blockade [7], has demonstrated success in treating a variety of cancers, including unresectable and metastatic cancers [8]. Nevertheless, only a fraction of patients can benefit from immunotherapy to date. Therefore, identification of predictive factors for immunotherapy is crucial. Some such factors have been identified, including tumor mutation burden (TMB) [9], PD-L1 expression [10,11], and deficient DNA mismatch repair [12,13]. Besides, the tumor immune microenvironment (TIME) is an important factor for response to immunotherapy [14]. The TIME can be simply classified as “cold” (without or lack of T-cell inflammation) and “hot” (with T-cell inflammation), depending on the production of pro-inflammatory cytokines and the level of T-cell infiltration [15]. It has been recognized that “hot” tumors respond better to immunotherapy than “cold” tumors [16]. Thus, distinguishing between “hot” and “cold” tumors may lead to optimal options for patients responding to immunotherapy. To distinguish between “hot” and “cold” ACCs, we performed hierarchical clustering of ACCs based on the enrichment of 23 immune-associated signatures to identify immune-specific subtypes of ACC. We demonstrated the stability and reproducibility of this classification in five independent cohorts. Furthermore, we analyzed subtype-specific molecular and clinical features, including the tumor microenvironment (TME), genomic features, pathways, phenotypes, and clinical outcomes. The identification of immune-specific subtypes of ACC may provide new insights into the pathogenesis and potential clinical implications for immunotherapy of this disease.

## 2. Materials and Methods

### 2.1. Datasets

From the genomic data commons (GDC) data portal (https://portal.gdc.cancer.gov/, accessed on 10 November 2020), we downloaded the data related to TCGA-ACC, including RNA-Seq gene expression profiles (RSEM-normalized, sample size: 79), somatic mutation profiles (whole exome sequence, sample size: 92), protein expression profiles (Reverse Phase Protein Array (RPPA)-normalized, sample size: 46), and clinical data (sample size: 92). From the NCBI Gene Expression Omnibus (GEO) (https://www.ncbi.nlm.nih.gov/geo/, accessed on 22 November 2020), we obtained four other ACC transcriptomic datasets: GSE10927 (Affymetrix Human Genome U133 Plus 2.0 Array, sample size: 33), GSE19750 (Affymetrix Human Genome U133 Plus 2.0 Array, sample size: 44), GSE90713 (Affymetrix Human Gene Expression Array, sample size: 58), and GSE143383 (GeneChip® PrimeView™ Human Gene Expression Array, sample size: 57). A summary of these datasets is shown in Supplementary Table S1.

### 2.2. Single-Sample Gene Set Enrichment Analysis

We used the “GSVA” R package [17] to perform the single-sample gene set enrichment analysis (ssGSEA). Based on gene expression profiles, the ssGSEA quantifies the enrichment level of a gene set in a sample. The ssGSEA score represents the degree to which the genes in the gene set are coordinately up- or down-regulated in the sample. We utilized the ssGSEA to evaluate the enrichment level of immune cells, biological processes, pathways, or phenotypic features based on the expression profiles of their marker or pathway genes. The marker or pathway genes are presented in Table S2.

### 2.3. Clustering Analysis

We hierarchically clustered ACCs based on the enrichment scores of 23 immune signatures. The 23 immune signatures included: cytokine and cytokine receptor (CCR), immune checkpoint molecules, cytolytic activity, human leukocyte antigen (HLA), inflammation-promoting, para-inflammation, T cell co-inhibition, T cell co-stimulation, tumor-infiltrating lymphocyte (TIL), activated CD8 T cell, central memory CD8 T cell, effector memory CD8 T cell, central memory CD4 T cell, T follicular helper cell, type 1 T helper cell, regulatory T cell, activated B cell, natural killer cell, myeloid-derived suppressor cell (MDSC), activated dendritic cell, macrophage, mast cell, and monocyte. The marker genes of these immune signatures are presented in Table S2. The hierarchical clustering first normalized the ssGSEA scores by Z-score and transformed them into distance matrices by the R function “dist” with the parameter: method = “Euclidean”. We ran the hierarchical clustering using the function “hclust” in the R package “Stats” with the parameters: method = “ward.D2” and members = NULL.

### 2.4. Prediction of the Immune-Specific Subtypes of ACC

To predict the immune subtypes of ACC by immune signatures, we first normalized the ssGSEA scores (attribute values) of the 23 immune signatures by Z-score. The Random Forest (RF) [18] classifier was used to perform class prediction. In the RF, we set 100 for the number of trees and took all 23 immune signatures as the attributes. We evaluated the classification performance using the accuracy and the weighted F-score. We performed the class prediction with Weka (version 3.8.5), a web tool for machine learning [19].

### 2.5. Survival Analysis

We compared overall survival (OS) and disease-free survival (DFS) rates between ACC subtypes using the Kaplan–Meier (K-M) method [20]. The K-M curves were utilized to show the survival rate differences, and the log-rank test was used to evaluate the significance of survival rate differences.

### 2.6. Evaluation of Tumor Immune Score, Stromal Score, and Intratumor Heterogeneity (ITH) Level

We used the ESTIMATE algorithm [21] to evaluate the tumor immune score and stromal score based on gene expression profiles. The immune score and stromal score represent the tumor immune infiltration level and stromal content, respectively. We used the DITHER algorithm [22] to evaluate ITH levels, which scores ITH at the DNA level based on the entropy of somatic mutations and copy number alterations (CNAs).

### 2.7. Evaluation of TMB and CNAs

TMB is the total count of non-synonymous somatic mutations in whole exons in the tumor, which was calculated with the input of the “maf” file. We obtained CNA (known as tumor aneuploidy) scores of TCGA-ACC from the publication by Knijnenburg et al. [23].

### 2.8. Logistic Regression Model

We used the logistic regression model to compare the contribution of TMB and CNA in predicting patients with high (>median) versus low (<median) activated CD8 T cell enrichment. We employed the R function “glm” to fit the binary model and the R function “lm.beta” in the R package “QuantPsyc” to calculate the standardized regression coefficients (β values) in the logistic regression analysis.

### 2.9. Pathway and Gene Ontology (GO) Analysis

We identified the KEGG [24] pathways highly enriched in Immunity-H and in Immunity-L, respectively. We first identified the differentially expressed genes (DEGs) using Student’s *t* tests with a threshold of false discovery rate (FDR) < 0.05 and fold change (FC) > 1.5. By inputting the upregulated genes in a subtype into the GSEA web tool [25], we obtained the KEGG pathways highly enriched in the subtype. Furthermore, we utilized the weighted gene co-expression network analysis (WGCNA) [26] to identify the gene modules enriched in the subtypes. Based on the expression correlations between the hub genes in gene modules, we displayed the representative GO traits to functional annotation.

### 2.10. Statistical Analysis

We used the Student’s *t* test (two-tailed) to compare two classes of normally distributed data, including gene expression levels, protein expression levels, and the ratios of two different immune signatures. In comparisons of two classes of non-normally distributed data, including ssGSEA scores of gene sets, TMB, CNA scores, immune scores, and stromal scores, we used the Mann–Whitney U test (one-tailed). We utilized Spearman’s method to evaluate correlations between two variables. The Fisher’s exact test was used to assess the correlation between two categorical variables. To adjust for *p*-values in multiple tests, we calculated FDR with the Benjamini and Hochberg method [27]. We performed all statistical analyses with the R programming language (version 3.6.0).

## 3. Results

### 3.1. Clustering Analysis Identifies Two Immune Subtypes of ACC

**Figure 1:**
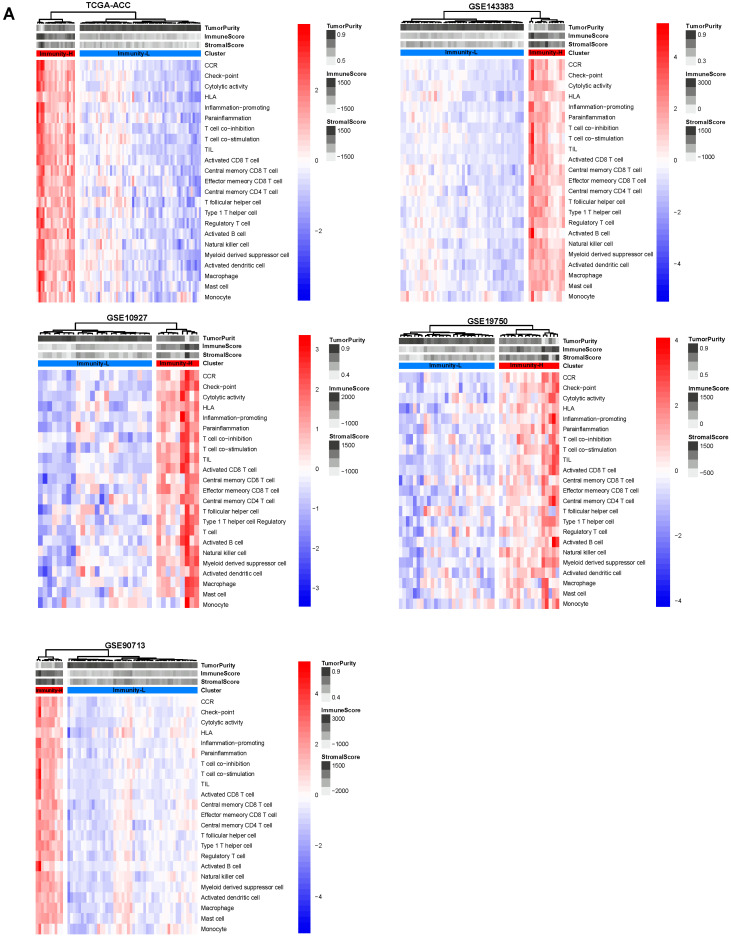

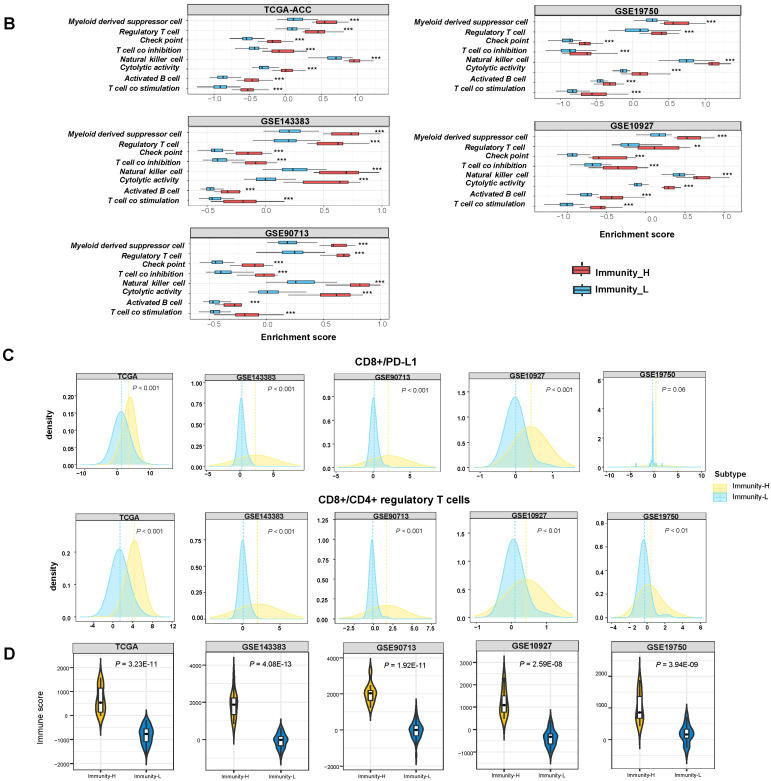

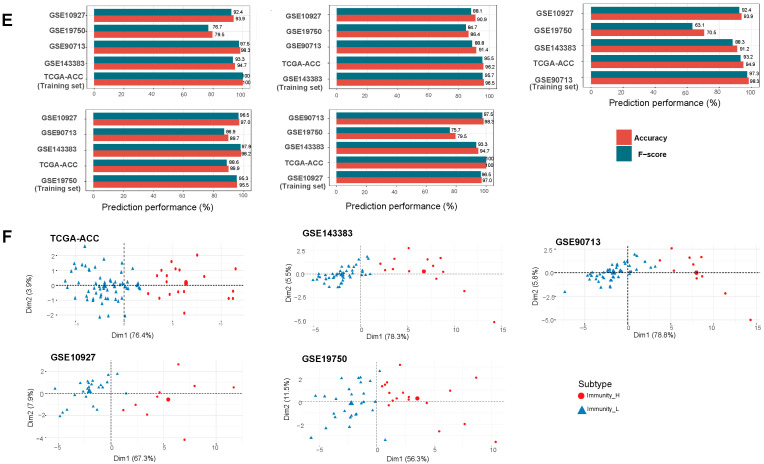
Hierarchical clustering of ACC tumors based on the enrichment of 23 immune signatures. (**A**) Clustering analyses uncovering two immune subtypes of ACC: Immunity-H and Immunity-L, which have high and low immune cell enrichment scores, respectively, consistently in five datasets. Comparisons of the enrichment scores of immunostimulatory signatures (T cell co-stimulation, activated B cell, cytolytic activity, and natural killer cell) and immunosuppressive signatures (T cell co-inhibition, immune checkpoint molecules, regulatory T cells, and myeloid-derived suppressor cells). (**B**) Ratios of immunostimulatory to immunosuppressive signatures (CD8+/PD-L1, CD8+/CD4+ regulatory T cells) between the two immune subtypes. (**C**) Immune scores evaluated by ESTIMATE [7]. (**D**) One-tailed Mann–Whitney U test (**B**,**D**) and Student’s *t* test (**C**) *p*-values are shown. ** *p* < 0.01, *** *p* < 0.001. It also applies to the following figures. (**E**) Prediction of the three immune subtypes of ACC by Random Forest based on the enrichment scores of 23 immune cell types. The 10-fold cross-validation results in the training set and prediction results in the other datasets are shown. (**F**) PCA confirms that ACCs can be clearly separated into two subgroups based on the ssGSEA scores of the immune signatures.

Based on the enrichment scores of 23 immune signatures representing diverse immune cell types or functions, we identified 2 immune subtypes of ACC consistently in 5 transcriptome datasets (TCGA-ACC, GSE143383, GSE90713, GSE19750, and GSE10927) (Figure 1A). Both subtypes, termed Immunity-H and Immunity-L, had high and low enrichment scores of these immune signatures, respectively. Notably, both immunostimulatory signatures (such as T cell co-stimulation, activated B cell, cytolytic activity, and natural killer cell) and immunosuppressive signatures (such as T cell co-inhibition, immune checkpoint molecules, regulatory T cells, and myeloid-derived suppressor cells) showed significantly higher enrichment levels in Immunity-H than in Immunity-L (one-tailed Mann–Whitney U test, *p* < 0.01) (Figure 1B). Nevertheless, the ratios of immunostimulatory/immunosuppressive signatures (CD8+/PD-L1 and CD8+/CD4+ regulatory T cells) were significantly higher in Immunity-H than in Immunity-L (two-tailed Student’s *t* test, *p* < 0.01) (Figure 1C). In addition, Immunity-H had higher immune scores than Immunity-L (one-tailed Mann–Whitney U test, *p* < 0.001) (Figure 1D). Taken together, these results suggest that Immunity-H has a stronger antitumor immune response than Immunity-L. Interestingly, *PD-L1* expression levels were also significantly higher in Immunity-H than in Immunity-L in TCGA-ACC (*p* < 0.001).

To verify whether the classification is predictable, we used one of the five datasets as the training set and the other four datasets as test sets, in turn, to predict both immune subtypes of ACC. Notably, the 10-fold cross-validation accuracies and weighted F-scores in training sets were all greater than 90%, and the prediction accuracies and weighted F-scores in test sets were mostly above 90% (Figure 1E). Furthermore, the principal component analysis confirmed that the ACCs could be clearly separated into two subgroups based on the enrichment scores of immune signatures (Figure 1F). Overall, these results demonstrate that the immunological classification of ACC is robust and predictable.

### 3.2. Immunity-H Has More Favorable Clinical Outcomes Than Immunity-L

**Figure 2:**
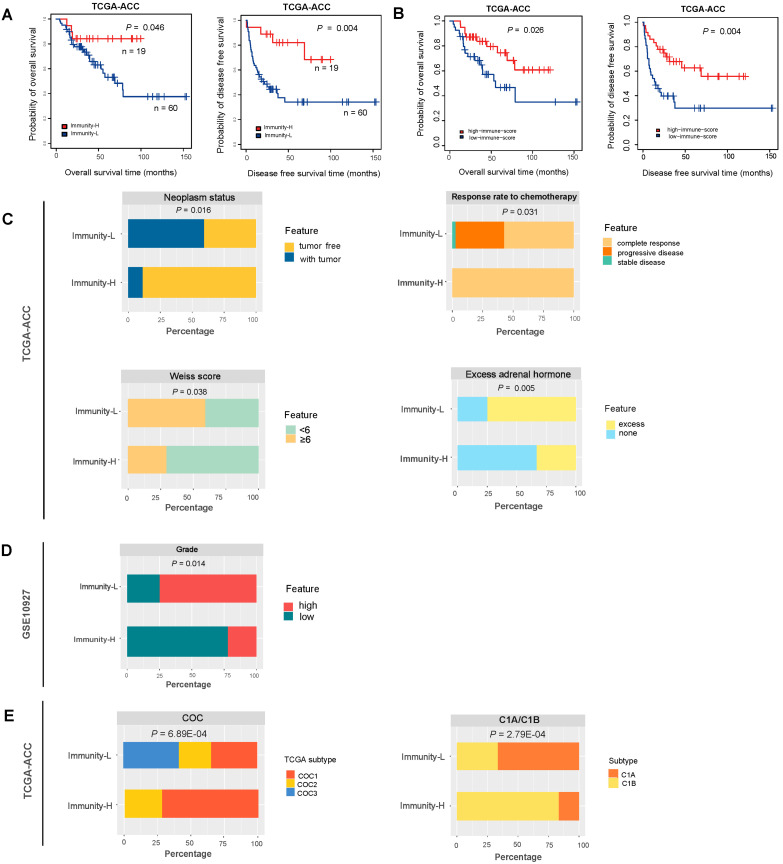
Comparisons of clinical outcomes between two immune subtypes of ACC. (**A**) Comparisons of overall survival (OS) and disease-free survival (DFS) rates between the immune subtypes by Kaplan–Meier curves. The log-rank test *p*-values are shown. (**B**) Comparisons of OS and DFS between high immune score (>median) and low immune score (<median). Comparisons of neoplasm status, the response rate to chemotherapy, Weiss score, and excess adrenal hormones, between the two immune subtypes in TCGA-ACC (**C**), and comparisons of grade between two immune subtypes in GSE10927 (**D**). The overlap between immune subtypes and CoC or C1A/C1B subtypes (**E**). The Fisher’s exact test *p*-values are shown (**C**–**E**).

We compared OS and DFS rates between both immune subtypes in TCGA-ACC, which had related data available. We found that Immunity-H displayed significantly higher OS and DFS rates than Immunity-L (log-rank test, *p* < 0.05) (Figure 2A). It indicates that the immune signature enrichment has a positive association with survival prognosis in ACC. Indeed, the patients with high immune scores (>median) showed better OS and DFS prognosis than the patients with low immune scores (<median) in TCGA-ACC (*p* = 0.026 and 0.004 for OS and DFS, respectively) (Figure 2B).

Moreover, in TCGA-ACC, we found that Immunity-H harbored a higher proportion of tumor-free patients than Immunity-L (89% versus 41%; Fisher’s exact test, *p* = 0.0003) (Figure 2C). Again, it suggested that Immunity-H had a better prognosis than Immunity-L. Furthermore, the response (complete response) rate to chemotherapy followed the pattern: Immunity-H (100%) > Immunity-L (58%), supporting the better prognosis in Immunity-H versus Immunity-L (Figure 2C). The Weiss score represents the extent of tumor progression by the pathological assessment, with a higher Weiss score indicating higher invasiveness of tumors [28]. We found that Immunity-H harbored a significantly lower proportion of tumors with Weiss score ≥6 than Immunity-L (29% versus 59%; *p* = 0.038) (Figure 2C). The excess adrenal hormones are a risk factor for ACC prognosis [29]. We found that Immunity-H involved a significantly lower proportion of tumors with excess adrenal hormones than Immunity-L (33% versus 75%; *p* = 0.005) (Figure 2C). In addition, in GSE10927, Immunity-H harbored a significantly lower proportion of high-grade tumors compared with Immunity-L (22% versus 75%; *p* = 0.013) (Figure 2D). Taken together, these results suggest that Immunity-H has better clinical outcomes than Immunity-L.

Based on a multi-omics analysis, the TCGA network identified three ACC subtypes in TCGA-ACC: CoC I, CoC II, and CoC III [29]. CoC I was highly enriched in immune-mediated pathways, and CoC III had the highest rate of disease progression and thus the worst prognosis. Interestingly, all CoC III patients were included in Immunity-L, suggesting that CoC III has low immunity. It is also in line with the fact that Immunity-L has a relatively poor prognosis among both immune subtypes. In contrast, more than 72% of Immunity-H patients belonged to CoC I, consistent with the high immunity in Immunity-H and CoC I (Figure 2E). In addition, based on gene expression profiles, the TCGA-ACC tumors were divided into two subgroups: C1A and C1B [29,30]. Among them, C1A was an aggressive subtype and C1B an indolent subtype. We found that around 67% of Immunity-L belonged to C1A as compared to 17% of Immunity-H. In contrast, around 33% of Immunity-L belonged to C1B versus 83% of Immunity-H. Again, it agrees with the worse prognosis in Immunity-L versus Immunity-H (Figure 2E).

### 3.3. Immunity-H has More Favorable Tumor Progression Phenotypes and Lower Levels of Genomic Instability Than Immunity-L

**Figure 3:**
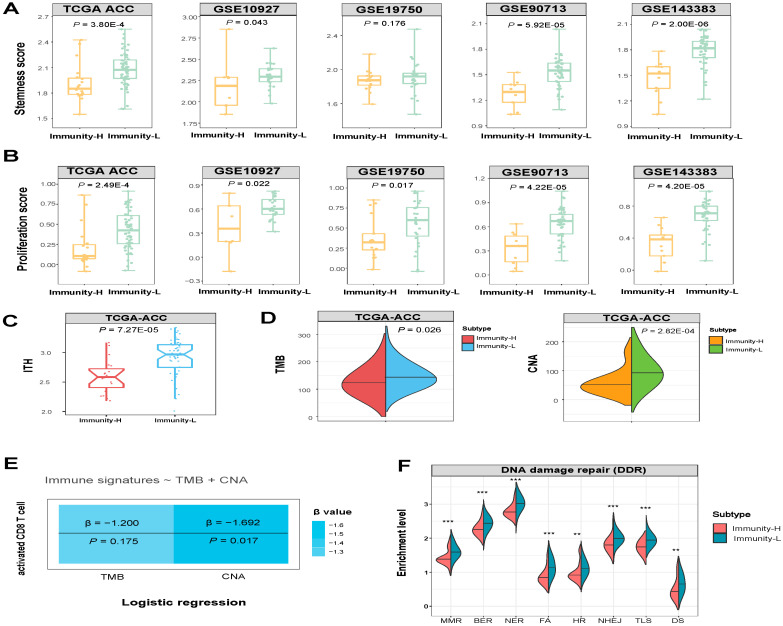
Comparisons of phenotypic and molecular features between the immune subtypes of ACC. Comparisons of the tumor stemness and proliferation (**A**,**B**), intratumor heterogeneity (ITH) (**C**), comparisons of CNA scores and TMB between the two ACC subtypes (**D**), and logistic regression models predicting high immune signature scores (>median) versus low immune signature scores (<median) of ACCs using CNA scores and TMB. CNA: copy number alteration. TMB: tumor mutation burden. Immune signature: activated CD8 T cell (**E**), comparisons of DNA damage repair (DDR) pathways (mismatch repair (MMR), base excision repair (BER), nucleotide excision repair (NER), Fanconi anemia (FA), homologous recombination (HR), non-homologous end joining (NHEJ), translesion DNA synthesis (TLS), and damage sensor (DS), *p*-values are shown. ** *p* < 0.01, *** *p* < 0.001 (**F**). The one-tailed Mann–Whitney U test *p*-values are shown in (**A**–**D**,**F**).

We compared several phenotypic features associated with tumor progression between Immunity-H and Immunity-L ACCs, including tumor stemness, proliferation potential, and ITH. Notably, Immunity-H displayed significantly lower stemness scores, proliferation potential, and ITH compared to Immunity-L (one-tailed Mann–Whitney U test, *p* < 0.05) (Figure 3A–C).

In TCGA-ACC, Immunity-H had significantly lower TMB and CNA scores than Immunity-L (one-tailed Mann–Whitney U test, *p* < 0.05) (Figure 3D). It indicated that Immunity-L had a higher level of genomic instability than Immunity-H. To compare the contribution of TMB and CNA in predicting antitumor immune responses in ACC, we used the logistic regression model to predict the activated CD8 T cell enrichment level with two predictors (TMB and CNA) in TCGA-ACC. Notably, CNA was a significant negative predictor of activated CD8 T cell enrichment (*p* = 0.017; β = −1.69), while TMB was not a significant predictor (*p* = 0.175) (Figure 3E). This result suggests that CNA has a stronger impact on antitumor immunity than TMB in ACC. It could explain why Immunity-L has a lower antitumor response than Immunity-H since Immunity-L has a higher level of CNA, which has been shown to correlate inversely with antitumor responses [31]. Furthermore, we compared the enrichment of nine major DNA damage repair (DDR) pathways between both subtypes. These pathways included mismatch repair, base excision repair, nucleotide excision repair, Fanconi anemia, homology-dependent recombination, non-homologous DNA end joining, direct damage reversal/repair, translesion DNA synthesis, and damage sensor. Notably, eight of the nine pathways displayed significantly higher enrichment in Immunity-L than in Immunity-H (one-tailed Mann–Whitney U test, *p* < 0.01) (Figure 3F). These results are justified since Immunity-L displays higher genomic instability than Immunity-H.

### 3.4. Identification of Genes with Significantly Different Mutation Rates between the Immune Subtypes of ACC

**Figure 4:**
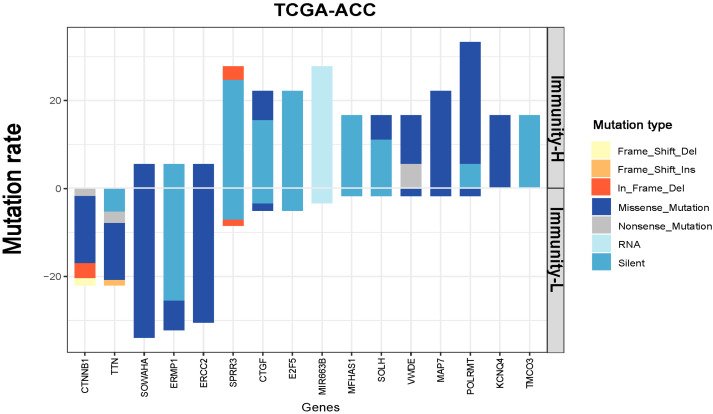
Different mutation rates between the immune subtypes of ACC. Eleven genes more frequently mutated in Immunity-H than in Immunity-L ACC (Fisher’s exact test, *p* < 0.05).

We observed 16 genes having significantly different mutation rates between Immunity-H and Immunity-L in TCGA-ACC (Fisher’s exact test, *p* < 0.05) (Figure 4). Of these genes, CTNNB1, TTN, SOWAHA, ERMP1, and ERCC2 showed significantly higher mutation rates in Immunity-L versus Immunity-H. Previous studies have shown that the CTNNB1 mutation may exert pro-tumorigenic function via activation of the Wnt/β-catenin pathway [32] and that ACCs overexpressing CTNNB1 have decreased antitumor immunity and poor prognosis [33,34]. It is consistent with a higher mutation rate of this gene in the subtype (Immunity-L) with a worse prognosis. ERCC2 encodes a protein functioning in regulating the nucleotide excision repair pathway [35]. Again, it is justified that this gene has a higher mutation rate in the subtype (Immunity-L) with higher genomic instability. In contrast, SPRR3, CTGF, E2F5, MIR663B, MFHAS1, SOLH, VWDE, MAP7, POLRMT, KCNQ4, and TMCO3 had significantly higher mutation rates in Immunity-H than in Immunity-L. A previous study has shown that POLRMT silencing or knockout can inhibit cell proliferation, migration, and invasion, and promote apoptosis [36]. It is consistent with the finding that this gene has a higher mutation rate in the subtype with higher immunity since apoptosis can promote antitumor immune responses [37].

### 3.5. Identification of Proteins Differentially Expressed between the Immune Subtypes of ACC

**Figure 5:**
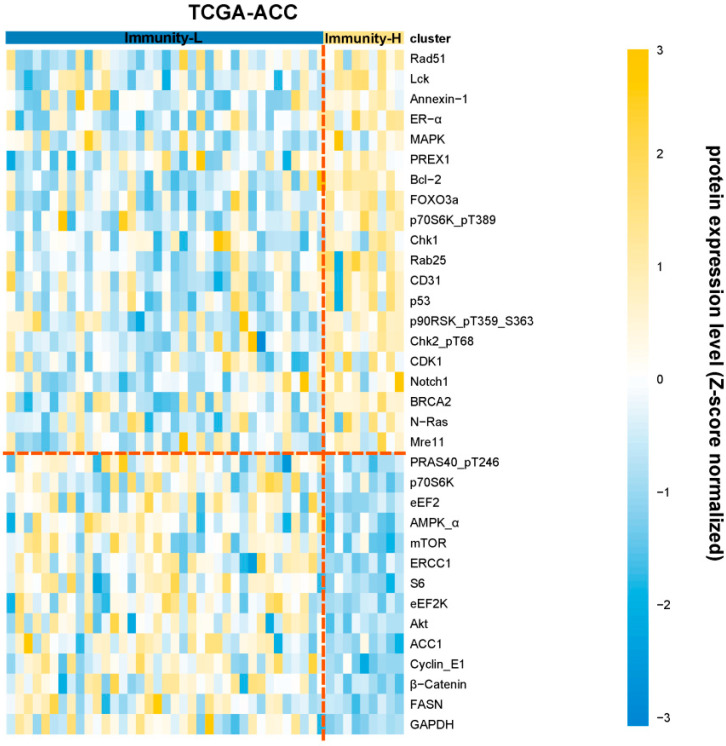
Differently expressed proteins between the immune subtypes of ACC. Heatmap showing the proteins with significant expression differences between Immunity-H and Immunity-L in TCGA-ACC (two-tailed Student’s *t* test, FDR < 0.05).

We compared the expression levels of 192 proteins between the immune subtypes of TCGA-ACC. Of them, 21 proteins were upregulated in Immunity-H relative to Immunity-L (two-tailed Student’s *t* test, FDR < 0.05) (Figure 5). These proteins included: Syk, Rad51, Lck, Annexin-1, ER-alpha, MAPK_pT202_Y204, PREX1, Bcl-2, FOXO3a, p70S6K_pT389, Chk1, Rab25, CD31, p53, p90RSK_pT359_S363, Chk2_pT68, CDK1, Notch1, BRCA2, N-Ras, and Mre11. In contrast, 14 proteins were upregulated in Immunity-L versus Immunity-H (Figure 5). These proteins included: PRAS40_pT246, p70S6K, eEF2, AMPK-α, mTOR, ERCC1, S6, eEF2K, Akt, ACC1, Cyclin_E1, β-catenin, FASN, and GAPDH.

### 3.6. Identification of Pathways and GO Enriched in the Immune Subtypes of ACC

**Figure 6:**
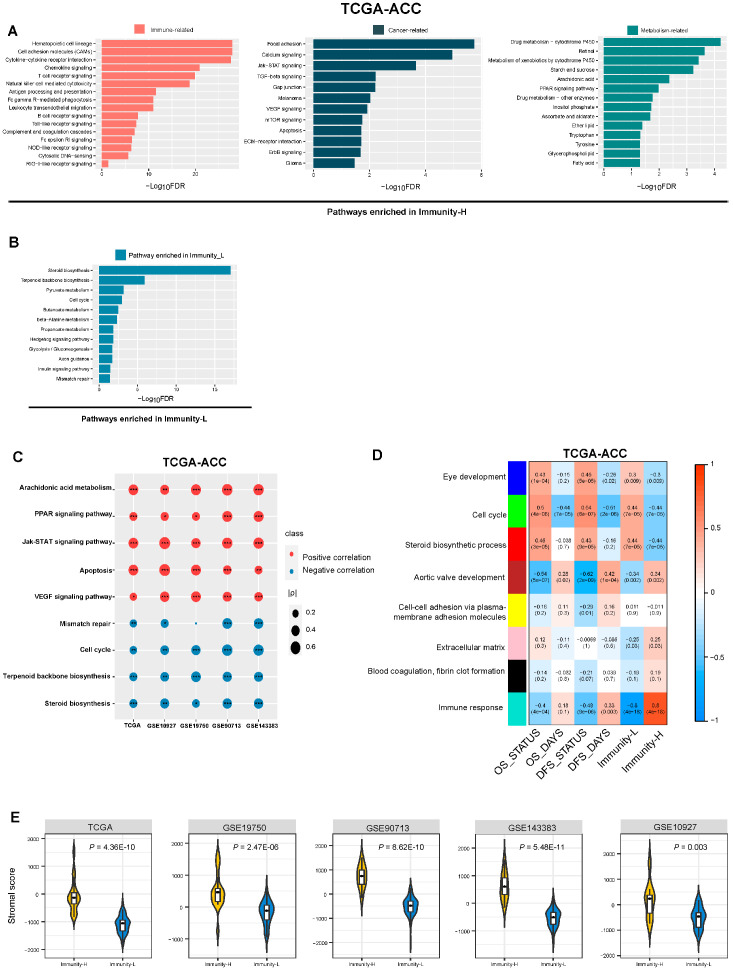
Pathways and gene ontology (GO) enriched in the immune subtypes of ACC. The KEGG pathways highly enriched in Immunity-H and Immunity-L in TCGA-ACC (**A**,**B**). (**C**) Spearman correlations between the enrichment scores of pathways upregulated in Immunity-H, Immunity-L, and immune scores in ACC. The correlation coefficients (*ρ*) and *p*-values are shown. * *p* < 0.5, ** *p* < 0.01, *** *p* < 0.001. (**D**) Eight gene modules that significantly differentiated ACC by subtype, survival time, or survival status identified by WGCNA. (**E**) Comparisons of stromal scores evaluated by ESTIMATE [7] between the immune subtypes of ACC (one-tailed Mann–Whitney U test, *p* < 0.01).

GSEA [25] identified pathways highly enriched in Immunity-H and Immunity-L in TCGA-ACC (Figure 6A). As expected, many immune-related pathways were enriched in Immunity-H, such as: hematopoietic cell lineage, T cell receptor signaling pathway, natural killer cell-mediated cytotoxicity, antigen processing and presentation, Fc gamma R-mediated phagocytosis, chemokine signaling pathway, cytokine–cytokine receptor interaction, B cell receptor signaling pathway, cytosolic DNA-sensing pathway, NOD-like receptor signaling pathway, complement and coagulation cascades, leukocyte transendothelial migration, Fc epsilon RI signaling pathway, Toll-like receptor signaling pathway, cell adhesion molecules (CAMs), and the RIG-I-like receptor signaling pathway. Meanwhile, we observed some cancer-related pathways highly enriched in Immunity-H, including the pathways of mTOR signaling, focal adhesion, calcium signaling, TGF-β signaling, gap junction, VEGF signaling, Jak-STAT signaling, ECM–receptor interaction, ErbB signaling, apoptosis, glioma, and melanoma. In addition, many metabolism-related pathways were upregulated in Immunity-H, including glycerophospholipid metabolism, ascorbate and aldarate metabolism, starch and sucrose metabolism, drug metabolism—cytochrome P450, retinol metabolism, metabolism of xenobiotics by cytochrome P450, inositol phosphate metabolism, drug metabolism—other enzymes, tryptophan metabolism, ether lipid metabolism, PPAR signaling, tyrosine metabolism, and fatty acid metabolism. As expected, many of the cancer- and metabolism-related pathways showed significant positive correlations of their enrichment scores with immune scores in the five datasets (Spearman’s correlation, *p* < 0.05) (Figure 6C). Furthermore, we identified many pathways enriched in Immunity-L, including pathways of steroid biosynthesis, terpenoid backbone biosynthesis, butanoate metabolism, insulin signaling pathway, mismatch repair, pyruvate metabolism, cell cycle, β-alanine metabolism, hedgehog signaling, propanoate metabolism, glycolysis/gluconeogenesis, and axon guidance (Figure 6B). As expected, many of these pathways showed significant negative correlations of their enrichment scores with immune scores in the five datasets (*p* < 0.05) (Figure 6C).

WGCNA [26] identified eight gene modules that significantly differentiated ACCs by survival status, survival time, and the immune subtypes (Figure 6D). Notably, six gene modules (indicated in blue, green, red, brown, pink, black, and turquoise, respectively) showed significantly different enrichment between Immunity-H and Immunity-L (*p* < 0.05). As expected, the immune response gene module (indicated in turquoise) had the strongest positive correlation with Immunity-H (R = 0.80; *p* = 4.00 × 10^−18^). Meanwhile, this module correlated negatively with OS and DFS survival status (OS: R = −0.40 and *p* = 4.00 × 10^−4^; DFS: R = −0.48 and *p* = 9.0 × 10^−6^). These results are consistent with the higher antitumor immune response in Immunity-H versus Immunity-L and the significant positive correlation between immune scores and survival prognosis in ACC. The extracellular matrix gene module (indicated in pink) was more highly enriched in Immunity-H versus Immunity-L (*p* = 0.03). It indicated that Immunity-H had more abundant stromal content than Immunity-L. Indeed, Immunity-H had significantly higher stromal scores than Immunity-L (*p* < 0.01) (Figure 6E). Consistent with the results from the pathway analysis, the cell cycle gene module was upregulated in Immunity-L relative to Immunity-H (*p* = 7.00 × 10^−5^). Additionally, this module correlated positively with OS and DFS status (OS: R = 0.50 and *p* = 4.00 × 10^−6^; DFS: R = 0.54 and *p* = 6.00 × 10^−7^). Again, the steroid biosynthetic process gene module was highly enriched in Immunity-L (*p* = 7 × 10^−5^). Additionally, this module had positive correlations with OS and DFS survival status (OS: R = 0.46 and *p* = 3.00 × 10^−5^; DFS: R = 0.43 and *p* = 9.00 × 10^−5^).

## 4. Discussion

**Figure 7:**
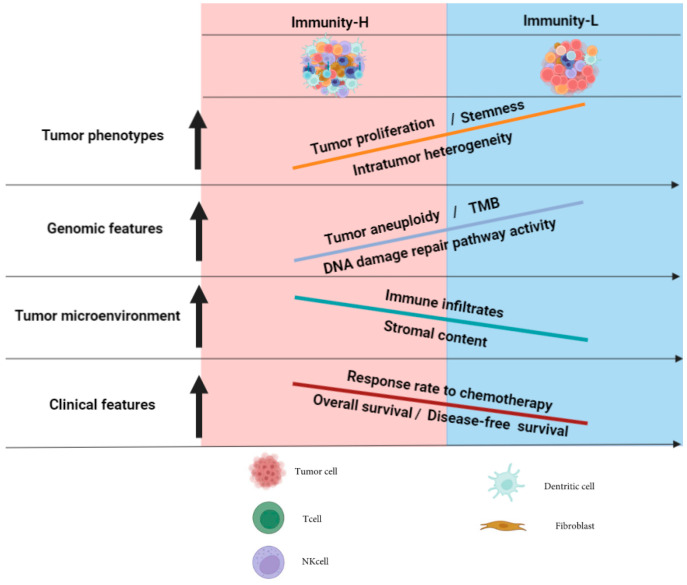
A summary of molecular and clinical characteristics of the immune subtypes of ACC. The figure was created with BioRender.com.

This study classified ACC based on the enrichment of 23 immune signatures and identified 2 immune-specific subtypes: Immunity-H and Immunity-L, which had high and low antitumor immunity, respectively. We demonstrated that this classification was reproducible and predictable by analyzing five different datasets. Furthermore, we showed that both subtypes had significantly different clinical and molecular characteristics. Compared with Immunity-H, Immunity-L had lower levels of immune cell infiltration, lower stromal content, worse overall and disease-free survival prognosis, lower response rate to chemotherapy, and higher tumor stemness, proliferation capacity, genomic instability, and ITH. Pathway and GO analysis showed that the immune, stromal, and apoptosis pathways were highly enriched in Immunity-H, while the cell cycle, steroid biosynthesis, and DDR pathways were highly enriched in Immunity-L (Figure 7).

Notably, the steroid biosynthetic process, which plays an immunosuppressive role in ACC [38], was prominently enriched in Immunity-L. In fact, ACC is an endocrine malignant tumor, often accompanied by spontaneous secretion of steroid hormones, including cortisol, sex hormones, and steroid precursors or aldosterone [39]. Previous studies have revealed that ACC patients with a high steroid phenotype have significantly weaker immune capabilities than those with a low steroid phenotype [40]. Many studies have indicated that excessive glucocorticoid hormones, including cortisol, result in immunosuppression by blocking the growth and maturation of immune cells and inhibiting activation and inducing apoptosis in lymphocytes [41,42]. In addition, glucocorticoids also block the function of peripheral T lymphocytes, leading to immune escape [42]. A recent immunotherapy clinical trial uncovered a pattern of immune resistance among cortisol-secreting ACCs (CS-ACCs) [43]. The CS-ACC patients showed a higher rate of immunotherapy failure compared to non-CS-ACC patients [44,45]. Therefore, ACC-induced hypercortisolism may be the main cause of “immune coldness” in some ACC patients.

Of the 21 proteins upregulated in Immunity-H, many function in the positive regulation of antitumor immune responses. For example, Syk as a tumor suppressor has a role in driving antitumor immune responses [46]. Lck incites antitumor immune responses by regulating T cell development [47]. CD31 encodes a protein that is a member of the immunoglobulin superfamily and is likely involved in leukocyte migration, angiogenesis, and integrin activation [48], supporting the higher tumor immunity in Immunity-H versus Immunity-L. PREX1 acts as a guanine nucleotide exchange factor for the RHO family of small GTP-binding proteins (RACs) to promote antitumor immune responses [49]. Furthermore, we observed that the most important tumor suppressor, p53, had significantly higher expression levels in Immunity-H than in Immunity-L. It conforms to previous findings that disfunction of p53 inhibits antitumor responses [37]. In addition, several protein kinases involved in signal transduction were included in the list of the 21 proteins, including MAPK and Lck. Of the 14 proteins upregulated in Immunity-L, β-catenin acts as a coactivator for transcription factors, leading to activation of Wnt-responsive genes to regulate cell adhesion [50]. The protein kinase mTOR acts as a central regulator of cellular metabolism, growth, and survival in response to hormones, growth factors, nutrients, and stress signals. The mTOR signaling pathway has been shown to be deregulated in many cancers [51]. Akt regulates many cancer-associated biological processes, including metabolism, proliferation, cell survival, growth, and angiogenesis [52]. Previous studies have revealed that AMPK plays a key role in modulating the response to immune checkpoint blockade and suggest that AMPK agonists may promote the efficacy of immunotherapy [53]. It supports our finding that AMPK is upregulated in “cold” tumors. The upregulation of these tumor invasion-associated proteins in Immunity-L could explain why Immunity-L had a worse prognosis than Immunity-H.

The pathway analysis supported the previous results. For example, the upregulation of steroid biosynthesis in Immunity-L indicated that steroids were more abundant in Immunity-L than in Immunity-H, consistent with the finding that the steroid phenotype is a risk factor for ACC [39]. The mismatch repair pathway was upregulated in Immunity-L versus Immunity-H, supporting the higher genomic instability in Immunity-L [54,55]. In addition, the upregulation of the cell cycle in Immunity-L reflected a higher cell proliferation potential in this subtype, consistent with previous results.

It is interesting to observe that Immunity-L has higher TMB than Immunity-H. It appears to conflict with the positive association between TMB and antitumor immune responses in many other cancers [56]. Nevertheless, our previous studies have revealed that the association between TMB and antitumor immune responses is cancer type-dependent [57]. That is, the association between TMB and antitumor immune responses could be positive or negative, depending on cancer types. Furthermore, Immunity-L has higher levels of CNA than Immunity-H. It is consistent with the negative effect of CNA on the antitumor immune infiltration [31]. Moreover, the logistic regression analysis demonstrated a stronger impact of CNA on antitumor immunity compared with TMB in ACC. Again, it supports previous findings that CNA has a stronger association with antitumor immune responses than TMB.

Previous studies have identified molecular subtypes of ACC, such as three subtypes identified by integration of multi-omics data in TCGA: CoC I, CoC II, and CoC III [29], and two subtypes defined by the gene expression profiles: C1A and C1B [30]. Our immune-specific subtyping demonstrated high overlaps with both CoC and C1A/B subtyping, with most of the aggressive C1A or CoC III patients belonging to Immunity-L. Although these previous subtyping methods have certain prognostic values, they have no translational relevance of targeted therapies or immunotherapy. In contrast, our immune-specific subtyping may provide clinical implications for immunotherapy recommendations in ACC. Meanwhile, we also observed significantly higher expression levels of FATE1 in Immunity-L than in Immunity-H. It is in line with previous studies showing that FATE1 expression is associated with increased steroidogenesis and reduced immune responses and thus is a robust prognostic indicator in ACC patients [58].

In ACC, the Weiss score is the most widely accepted pathological system for classifying adrenocortical tumors (ACTs) as benign (adrenocortical adenoma (ACA)) or malignant (adrenocortical carcinoma (ACC)). A total score of ≤2 indicates an ACA, while a score of ≥3 is indicative of an ACC [55]. In addition, previous studies have identified multiple genes and pathways as potential prognostic biomarkers for patients with ACC, such as the driver genes IGF2, TP53, CTNNB1 [6], NR5A1, and FSCN1 [59,60], and the p53-RB and WNT-β-catenin pathways [56]. Hyperactivation of the β-catenin pathway and loss of p53 function are potential intrinsic tumor drivers in ACC [57]. Increased secretion of steroids also contributes to the immunosuppressive microenvironment in ACC [36]. Both NR5A1 and FSCN1 are adverse prognostic factors in ACC [59,60]. Notably, both genes displayed significantly higher expression levels in Immunity-L than in Immunity-H in all five datasets, in agreement with the worse prognosis in Immunity-L versus Immunity-H. Although surgery, chemotherapy, and radiotherapy are currently being used to treat ACC, these treatment strategies are often not effective for unresectable or metastatic ACC patients [58]. On the other hand, immunotherapy has achieved success in treating many unresectable or metastatic cancers, including ACC [41,42,59]. Thus, the new immune-based classification has the potential to identify the patients more suitable for immunotherapy to achieve better clinical outcomes.

This study has several limitations. First, because ACC is a rare cancer, the sample size of ACC patients is relatively small. Second, we obtained the results by bioinformatics analysis, but lacked experimental validation. Third, although our classification has potential value in stratifying ACC patients for immunotherapy, a further validation of its clinical applicability is needed.

## 5. Conclusions

This study demonstrated the stability and reproducibility of immune-specific subtyping of ACC. The subtype with high immunity had a better survival prognosis, higher response rate to chemotherapy, and lower tumor stemness, proliferation capacity, genomic instability, and ITH, compared to the subtype with low immunity. This analysis provides new insights into the tumor biology as well as potential clinical values for the management of this disease.

## Data Availability

The datasets analyzed during the current study are available from the corresponding author upon reasonable request.

## Supplementary Materials

The following supporting information can be downloaded at: https://www.mdpi.com/article/10.3390/biom13010104/s1, Table S1: A summary of the datasets analyzed. Table S2: The gene sets representing immune signatures, pathways, and biological processes.
