# Supplementary material for: Identifying Immune-Specific Subtypes of Adrenocortical Carcinoma Based on Immunogenomic Profiling
Source: Biomolecules. 2023 Jan 4;13(1):104. doi: 10.3390/biom13010104 (PMC9855412; doi:10.3390/biom13010104)
Supplement: Supplementary file 1 [file biomolecules-13-00104-s001.zip › biomolecules-1984375-supplementary.pdf]

biomolecules

MDPI

Table S1. A summary of the datasets analyzed.

Dataset	Number of Samples	Source
TCGA-ACC	79	https://portal.gdc.cancer.gov/projects/TCGA-ACC
GSE90713	58	https://www.ncbi.nlm.nih.gov/geo/query/acc.cgi?acc=GSE90713
GSE10927	33	https://www.ncbi.nlm.nih.gov/geo/query/acc.cgi?acc=GSE10927
GSE19750	44	https://www.ncbi.nlm.nih.gov/geo/query/acc.cgi?acc=GSE19750
GSE143383	57	https://www.ncbi.nlm.nih.gov/geo/query/acc.cgi?acc=GSE143383

Table S2. The gene sets represent immune signatures, pathways, and biological processes.

**23 immune signatures for clustering ACC**	Activated CD8 T cell	*ADRM1, AHSA1, C1GALT1C1, CCT6B, CD37, CD3D, CD3E, CD3G, CD69, CD8A, CETN3, CSE1L, GEMIN6, GNLY, GPT2, GZMA, GZMH, GZMK, IL2RB, LCK, MPZL1, NKG7, PIK3IP1, PTRH2, TIMM13, ZAP70*
	Central memory CD8 T cell	*ACTN4, ADAM12, ADCY9, F13A1, FCER1G, FCGR3B, FGF7, FKBP4, GLUD1, GM2A, GUSB, IL1RN, NOL11, NTRK1, RARA, RNF128, SIGLEC1, TNFRSF11A, TOX4, UBA52, ULBP1*
	Effector memory CD8 T cell	*ACAP1, APOL3, ARHGAP10, ATP10D, C3AR1, CCR5, CD160, CD55, CFLAR, CMKLR1, DAPP1, FCRL6, FLT3LG, GZMM, HAPLN3, HLA-DMB, HLA-DPA1, HLA-DPB1, IFI16, LIME1, LTK, NFKBIA, SETD7, SIK1, TRIB2*
	CCR	*CCL16, TPO, TGFBR2, CXCL2, CCL14, TGFBR3, IL11RA, CCL11, IL4I1, IL33, CXCL12, CXCL10, BMPER, BMP8A, CXCL11, IL21R, IL17B, TNFRSF9, ILF2, CX3CR1, CCR8, TNFSF12, CSF3, TNFSF4, BMP3, CX3CL1, BMP5, CXCR2, TNFRSF10D, BMP2, CXCL14, CCL28, CXCL3, BMP6, CCL21, CXCL9, CCL23, IL6, TNFRSF18, IL17RD, IL17D, IL27, CCL7, IL1R1, CXCR4, CXCR2P1, TGFB1I1, IFNGR1, IL9R, IL1RAPL1, IL11, CSF1, IL20RA, IL25, TNFRSF4, IL18, ILF3, CCL20, TNFRSF12A, IL6ST, CXCL13, IL12B, TNFRSF8, IL6R, BMPR2, IFNE, IL1RAPL2, IL3RA, BMP4, CCL24, TNFSF13B, CCR4, IL2RA, IL32, TNFRSF10C, IL22RA1, BMPR1A, CXCR5, CXCR3, IF8, IL17REL, IFNB1, IFR1, TNFRSF1B, CCL17, IFNL1, IL16, IL1RL1, ILK, CCL25, ILDR2, CXCR1, IL36RN, IL34, TGFB1, IFNG, IL19, ILKAP, BMP2K, CCR10, ILDR1, EPO, CCR7, IL17C, IL23A, CCR5, IL7, EPOR, CCL13, IL2RG, IL31RA, TNFAIP6, IFNL2, BMP1, IL12RB1, TNFAIP8, IL4R, TNFRSF6B, TNFAIP8L1, TNFRSF10B, IFNL3, CCL5, CXCL6, CXCL1, CCR3, TNFSF11, CSF1R, IL21, IL1RAP, IL12RB2, CCL1, IL17RA, CCR1, IL1RN, TNFRSF11B, TNFRSF14, IL13, IL2RB, BMP8B, CCL2, IL24, IL18RAP, TGFBI, TNFSF10, TNFRSF11A, CXCL5, IL5RA, TNFSF9, IL1RL2, TNFRSF13C, IL36G, IL15RA, TNFRSF21, CXCL8, IL22RA2, TNFAIP8L2, IL18R1, IFNLR1, CXCR6, CCL3L3, TNFRSF1A, IL17RE, IFNGR2, IL17RC, TNFAIP8L3, ILVBL, TGFBRAP1, CCL4L1, CSF2RA, NOCT, CCL26, TNFAIP1, CCRL2, IF10, TNFRSF17, IF13, IL20, IL18BP, CCL3L1, TNFSF12-TNFSF13, IL5, IL23R, IL26, TNF, TGFA, CSF2, IL1F10, CXCL17, TNFSF13, IF4, IL37, IL12A, IL7R, IF1, IL1A, IL4, IL2, CCL22, CSF3R, IL10, IFNK, TGFB2, IL1R2, IL1B, IL17F, IL27RA, IL15, TNFSF8, IL36B, XCL1, CXCL16, TNFRSF19, IL3, CCL3, IF2, BMPR1B, IF21, TNFSF18, CCL8, IL17RB, TNFRSF25, IL22, IL10RB, IFR2, CCL18, IF16, CSF2RB, IL36A, TNFAIP3, IL13RA2, IL13RA1, CCR9, TNFRSF10A, IF7, IFNW1, XCL2, TNFSF14, CCR2, BMP15, BMP10, CCL15-CCL14, TGFBR1, IF5, BMP7, IF14, IL20RB, IL10RA, IF17, CCR6,*

	TGFB3, CCL15, CCL4, CCL27, TNFRSF13B, TNFAIP2, IL31, IL17A, TNFSF15, CCL19, IF6, IL9
Central memory CD4 T cell	*ABHD3, AHNAK, ANXA2P2, AQP3, ATHL1, BMI1, BZW2, CD63, COL4A1, CYLD, ELMO2, FYN, GLIPR1, GSS, IFITM2, ITGB1, ITGB2, KLF5, LSP1, NDUFB9, PKM2, SFXN3, SIRPG, SMAD4, STX4, TRADD*
Check-point	*IDO1, LAG3, CTLA4, TNFRSF9, ICOS, CD80, PDCD1LG2, TIGIT, CD70, TNFSF9, ICOSLG, KIR3DL1, CD86, PDCD1, LAIR1, TNFRSF8, TNFSF15, TNFRSF14, IDO2, CD276, CD40, TNFRSF4, TNFSF14, HHLA2, CD244, CD274, HAVCR2, CD27, BTLA, LGALS9, TMIGD2, CD28, CD48, TNFRSF25, CD40LG, ADORA2A, VTCN1, CD160, CD44, TNFSF18, TNFRSF18, BTNL2, VSIR, CD200R1, TNFSF4, CD200, NRP1*
T follicular helper cell	*B3GAT1, CDK5R1, PDCD1, BCL6, CD200, CD83, CD84, FGF2, GPR18, CEBPA, CECR1, CLEC10A, CLEC4A, CSF1R, CTSS, DMN, DPP4, LRRC32, MC5R, MICA, NCAM1, NCR2, NRP1, PDCD1LG2, PDCD6, PRDX1*
Cytolytic activity	*PRF1, GZMA*
Type 1 T helper cell	*CD70, TBX21, ADAM8, AHCYL2, ALCAM, B3GALNT1, BBS12, BST1, CD151, CD47, CD48, CD52, CD53, CD59, CD6, CD68, CD7, CD96, CFHR3, CHRM3, CLEC7A, COL23A1, COL4A4, COL5A3, DAB1, DLEU7*
HLA	*HLA-E, HLA-DPB2, HLA-C, HLA-J, HLA-DQB1, HLA-DQB2, HLA-DQA2, HLA-DQA1, HLA-A, HLA-DMA, HLA-DOB, HLA-DRB1, HLA-H, HLA-B, HLA-DRB5, HLA-DOA, HLA-DPB1, HLA-DRA, HLA-DRB6, HLA-L, HLA-F, HLA-G, HLA-DMB, HLA-DPA1*
Inflammation-promoting	*CCL5, CD19, CD8B, CXCL10, CXCL13, CXCL9, GNLY, GZMB, IFNG, IL12A, IL12B, IRF1, PRF1, STAT1, TBX21*
Regulatory T cell	*CCL3L1, CD72, CLEC5A, FOXP3, ITGA4, L1CAM, LIPA, LRP1, LRRC42, MARCO, MMP12, MNDA, MRC1, MS4A6A, PELO, PLEK, PRSS23, PTGIR, ST8SIA4, STAB1*
Activated B cell	*ADAM28, CD180, CD79B, BLK, CD19, MS4A1, TNFRSF17, IGHM, GNG7, MICAL3, SPIB, HLA-DOB, IGKC, PNOC, FCRL2, BACH2, CR2, TCL1A, AKNA, ARHGAP25, CCL21, CD27, CD38, CLEC17A, CLEC9A, CLECL1*
Parainflammation	*CXCL10, PLAT, CCND1, LGMN, PLAUR, AIM2, MMP7, ICAM1, MX2, CXCL9, ANXA1, TLR2, PLA2G2D, ITGA2, MX1, HMOX1, CD276, TIRAP, IL33, PTGES, TNFRSF12A, SCARB1, CD14, BLNK, IFIT3, RETNLB, IFIT2, ISG15, OAS2, REL, OAS3, CD44, PPARG, BST2, OAS1, NOX1, PLA2G2A, IFIT1, IFITM3, IL1RN*
T cell co-inhibition	*BTLA, VSIR, CD160, CD244, CD274, CTLA4, HAVCR2, LAG3, LAIR1, TIGIT*
Natural killer cell	*AKT3, AXL, BST2, CDH2, CRTAM, CSF2RA, CTSZ, CXCL1, CYTH1, DAXX, DGKH, DLL4, DPYD, ERBB3, F11R, FAM27A, FAM49A, FASLG, FCGR1A, FN1, FSTL1, FUCA1, GBP3, GLS2, GRB2, LST1*
T cell co-stimulation	*CD2, CD226, CD27, CD28, CD40LG, ICOS, SLAMF1, TNFRSF18, TNFRSF25, TNFRSF4, TNFRSF8, TNFRSF9, TNFSF14*
TIL	*ITM2C, CD38, THEMIS2, GLYR1, ICOS, F5, TIGIT, KLRD1, IRF4, PRKCQ, FCRL5, SIRPG, LPXN, IL2RG, CCL5, LCK, TRAF3IP3, CD86, MAL, LILRB1, DOK2, CD6, PAG1, LAX1, PLEK, PIK3CD, SLAMF1, XCL1, GPR171, XCL2, TBX21, CD2, CD53, KLHL6, SLAMF6, CD40, SIT1, TNFRSF4, CD79A, CD247, LCP2, CD3D, CD27, SH2D1A, FYB1, ARHGAP30, ACAP1, CST7, CD3G, IL2RB, CD3E, FCRL3, CORO1A, ITK, TCL1A, CYBB, CSF2RB, IKZF1, NCF4, DOCK2, CCR2, PTPRC, PLAC8, NCKAP1L, IL7R, SEPTIN6, CD28, STAT4, CD8A, LY9, CD48, HCST, PTPRCAP, SASH3, ARHGAP25, LAT, TRAT1, IL10RA, PAX5, CCR7, DOCK11, PARVG, SPNS1, CD52, HCLS1, ARHGAP9, GIMAP6, PRKCB, MS4A1, GPR18, TBC1D10C, GVINP1, P2RY8, EVI2B, VAMP5, KLRK1, SELL,*

		MPEG1, MS4A6A, ARHGAP15, MFNG, GZMK, SELPLG, TARP, GIMAP7, RIPOR2, INPP5D, ITGA4, MZB1, GPSM3, STK10, CLEC2D, IL16, NLRC3, GIMAP5, GIMAP4, IFFO1, CFH, PVRIG, CFHR1
	Myeloid derived suppressor cell	*CCR2, CD14, CD2, CD86, CXCR4, FCGR2A, FCGR2B, FCGR3A, FERMT3, GPSM3, IL18BP, IL4R, ITGAL, ITGAM, PARVG, PSAP, PTGER2, PTGES2, S100A8, S100A9*
	Activated dendritic cell	*ABCD1, C1QC, CAPG, CCL3L3, CD207, CD302, ATP5B, ATP5L, ATP6V1A, BCL2L1, C1QB, SNURF, SPCS3, CCNA1, CEACAM8, NOS2, SRA1, TNFRSF6B, TREM1, TREML1, RHOA, SLC25A37, TNFSF14, TREML4, VNN2, XPO6*
	Macrophage	*AIF1, CCL1, CCL14, CCL23, CCL26, CD300LB, CNR1, CNR2, EIF1, EIF4A1, FPR1, FPR2, FRAT2, GPR27, GPR77, RNASE2, MS4A2, BASP1, IGSF6, HK3, VNN1, FES, NPL, FZD2, FAM198B, HNMT*
	Mast cell	*ADAMTS3, CPA3, CMA1, CTSG, ARHGAP15, CPM, FCN1, FTL, HSPA6, ITGA9, RNASE3, S100A4, SIGLEC8, SLC6A4, PTGS2, EGR3, PILRA*
	Monocyte	*ASGR2, CFP, ASGR1, CD1D, UPK3A, ACTG1, ANXA5, ATP6V1B2, CFL1, DAZAP2, CTBS, EMR4P, HIVEP2, MARCKSL1, MBP, MMP15, PNPLA6, TMBIM6, PQBP1, TEX264, IKZF1*
**DNA damage repair (DDR) pathways**	Base excision repair (BER)	*NEIL2, MPG, SMUG1, XRCC1, POLE4, HMGB1, POLE3, POLD4, MBD4, OGG1, UNG, POLD3, PCNA, NEIL1, POLE2, PARP4, PARP3, PARP2, POLB, APEX1, POLL, POLD1, POLD2, POLE, NEIL3, TDG, APEX2, LIG3, HMGB1P1, NTHL1, HMGB1P40, FEN1, LIG1, MUTYH, PARP1*
	Nucleotide excision repair (NER)	*MNAT1, POLE4, ERCC4, POLE3, ERCC3, ERCC6, ERCC5, GTF2H5, POLD4, ERCC2, RFC4, CETN2, RFC5, RPA1, RAD23B, RBX1, DDB2, RPA3, POLD3, RPA2, RAD23A, PCNA, RPA4, DDB1, POLE2, ERCC1, POLD1, POLD2, POLE, RFC1, RFC3, RFC2, XPC, XPA, GTF2H2, GTF2H1, CDK7, LIG1, CUL4A, CUL4B, ERCC8, CCNH, GTF2H4, GTF2H3*
	Mismatch repair (MMR)	*MLH3, POLD1, MLH1, POLD2, RFC1, MSH2, RFC3, RFC2, MSH3, POLD4, PMS2, RFC4, LIG1, RFC5, RPA1, MSH6, RPA3, POLD3, RPA2, PCNA, SSBP1, RPA4, EXO1*
	Fanconi anemia (FA)	*ATRIP, ATR, FANCM, FAAP24, CENPS, CENPS-CORT, CENPX, TELO2, HES1, FAAP100, FANCA, FANCB, FANCC, FANCE, FANCF, FANCG, FANCL, WDR48, USP1, UBE2T, FANCI, FANCD2, BRCA2, PALB2, RAD51C, RAD51, BRCA1, BRIP1, FAN1, MLH1, PMS2, REV1, REV3L, POLH, POLI, POLK, POLN, RMI1, RMI2, TOP3A, TOP3B, BLM, RPA1, RPA2, RPA3, RPA4, MUS81, EME1, EME2, ERCC4, ERCC1, SLX1A, SLX1B, SLX4*
	homology-dependent recomination (HR)	*RAD54L, XRCC3, RAD51B, NBN, RAD52, RAD51D, XRCC2, POLD4, BLM, EME1, RPA1, RAD51, POLD3, RAD50, RPA3, RPA2, RPA4, RAD51C, MRE11, BRCA2, POLD1, POLD2, MUS81, TOP3A, RAD54B, SEM1, TOP3B, SSBP1*
	non-homologous DNA end joining (NHEJ)	*XRCC6, XRCC5, DCLRE1C, PRKDC, POLL, POLM, DNTT, LIG4, XRCC4, NHEJ1, RAD50, MRE11, FEN1*
	Direct damage reversal/repair (DR)	*MGMT, ALKBH2, ALKBH3, ASCC3*
	Translesion DNA synthesis (TLS)	*POLB, POLM, UBE2A, PCNA, HLTF, MAD2L2, POLH, POLI, POLK, POLN, POLQ, RAD18, REV1, REV3L, SHPRH, UBE2B, UBE2N, UBE2V2, USP1, WDR48*
	Damage sensor (DS)	*NUDT1, NUDT15, NUDT18, RRM1, RRM2*
**Biological processes**	stem cell-associated	*DNMT3B, PFAS, XRCC5, HAUS6, TET1, IGF2BP1, PLAA, TEX10, MSH6, DLGAP5, MTREX, SOHLH2, RRAS2, PAICS, CPSF3, LIN28B, IPO5, BMPR1A, ZNF788P, ASCC3, FANCB, HMGA2, TRIM24, ORC1, HDAC2,*

		HESX1, INHBE, MIS18A, DCUN1D5, MRPL3, CENPH, MYCN, HAUS1, GDF3, TBCE, RIOK2, BCKDHB, RAD1, NREP, ADH5, PLRG1, ROR1, RAB3B, DIAPH3, GNL2, FGF2, NMT2, KIF20A, CENPI, DDX1, XXYLT1, GPR176, BBS9, RTRAF, BOD1, CDC123, SNRPD3, FAM118B, DPH3, EIF2B3, RPF2, APLP1, DACT1, PDHB, C14orf119, DTD1, SAMM50, CCL26, MED20, UTP6, RARS2, ARMCX2, RARS, MTHFD2, DHX15, HTR7, MTHFD1L, ARMC9, XPOT, IARS, HDX, ACTRT3, ERCC2, TBC1D16, GARS, KIF7, UBE2K, SLC25A3, ICMT, UGGT2, ATP11C, SLC24A1, EIF2AK4, GPX8, ALX1, OSTC, TRPC4, HAS2, FZD2, TRNT1, MMADHC, SNX8, CDH6, HAT1, SEC11A, DIMT1, TM2D2, FST, GBE1
	Proliferation	*CCNB1, CDC20, CDKN3, CDK1, MAD2L1, PRC1, RRM2, MKI67*
